# ^177^Lu‐Gold Nanohybrids in Radiotherapeutic Approaches Against Cancer

**DOI:** 10.1002/smsc.202400550

**Published:** 2024-12-12

**Authors:** Mathilde Ponchelle, Marek Pruszyński, Edmond Gravel, Eric Doris

**Affiliations:** ^1^ Université Paris‐Saclay, CEA, INRAE Département Médicaments et Technologies pour la Santé (DMTS), SCBM 91191 Gif‐sur‐Yvette France; ^2^ NOMATEN Centre of Excellence National Centre for Nuclear Research 05‐400 Otwock Poland; ^3^ Centre of Radiochemistry and Nuclear Chemistry Institute of Nuclear Chemistry and Technology 03‐195 Warsaw Poland

**Keywords:** gold nanoparticles, lutetium‐177, nanomedicines, radioenhancement, radiopharmaceuticals

## Abstract

Internal radionuclide therapy using lutetium‐177 (^177^Lu)‐based radiopharmaceuticals has emerged as a promising approach in the field of nuclear medicine and oncology. Combining the radioactive lanthanide with high atomic number metal nanoparticles such as gold potentiates the radiotoxicity of lutetium by an amplification process. ^177^Lu‐gold nanohybrids have been investigated by various research groups, both in vitro and in vivo, to decipher the physicochemical, radiochemical, and biological factors that would enable selective and optimal dose deposition in tumors. This review focuses on ^177^Lu‐gold tailored approaches that are developed in the literature to locally enhance internal radiotherapeutic effects.

## Graphical abstract

This article reviews the literature on nanoscale systems incorporating radioactive lutetium‐177 and gold nanoparticles as amplifying units, applied to internal radionuclide therapy against cancer. A presentation of the lutetium radionuclide, the radioenhancing effect of gold nanoparticles, tumor delivery strategies, and the state of the literature on the design and use of ^177^Lu‐gold nanohybrids in cancer therapy are detailed.

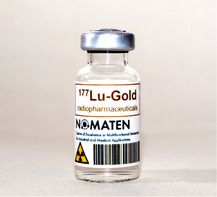

## 1 Introduction

Cancer is a major public health issue and one of the leading causes of mortality worldwide. Depending on the type of cancer and stage of the disease, a number of therapeutic regimens can be considered, including systemic (chemotherapy, immunotherapy, or hormone therapy) and local treatments. Among the local therapies, while surgery is the mainstay for patients with resectable tumors, radiotherapy is applied to about half of all patients, either as monotherapy or as part of multimodal cancer treatments. In radiotherapeutic approaches, most often delivered in the form of photon beams from a source outside the body (e.g., X‐rays or gamma‐rays), the interaction of the radiation with the biological medium induces the generation of reactive oxygen species (ROS) that are capable of altering not only nucleic acids of genetic material but also nearby membrane lipids and cytosolic proteins. The formation of oxidized adducts results in dysfunctions of the cellular machinery, ultimately leading to cell death. Although focused, external radiotherapy lacks selectivity and cannot discriminate between healthy and cancerous tissues, which can lead to adverse side effects. On the other hand, internal radioisotope therapy consists of introducing the source of irradiation directly into the body. The irradiation source is typically a radioactive element emitting short‐range ionizing radiations such as β^−^ or α‐emission. It can be either in the solid state and introduced in the immediate vicinity of the tumor (brachytherapy), or in the liquid state and administered either orally or intravenously (systemic radiotherapy). Systemic radiation therapy with radioactive iodine can be used, for example, to treat certain thyroid cancers. Internal radionuclide therapy ensures that the radiation dose level is high at the tumor site, with minimal adverse effects on the surrounding healthy tissues due to the short range of irradiation. More generally, if precision medicine is to be achieved, targeted radionuclides could provide an appropriate response to the treatment of cancers, including metastatic ones. Tumor‐targeting properties can be imparted to the radionuclide by associating it with a carrier system capable of selectively reaching the affected area in vivo. These include nanoscale carriers^[^ ^1^ ^]^ such as organic/inorganic nanoparticles,^[^ ^2^ ^]^ ligands/antibodies,^[^ ^3^ ^]^ or a combination of both. In addition, the association with metallic nanoparticles of high atomic number (e.g., gold) can potentiate radiotherapy by a radioenhancement effect.^[^ ^4^ ^]^

**Figure 1:**
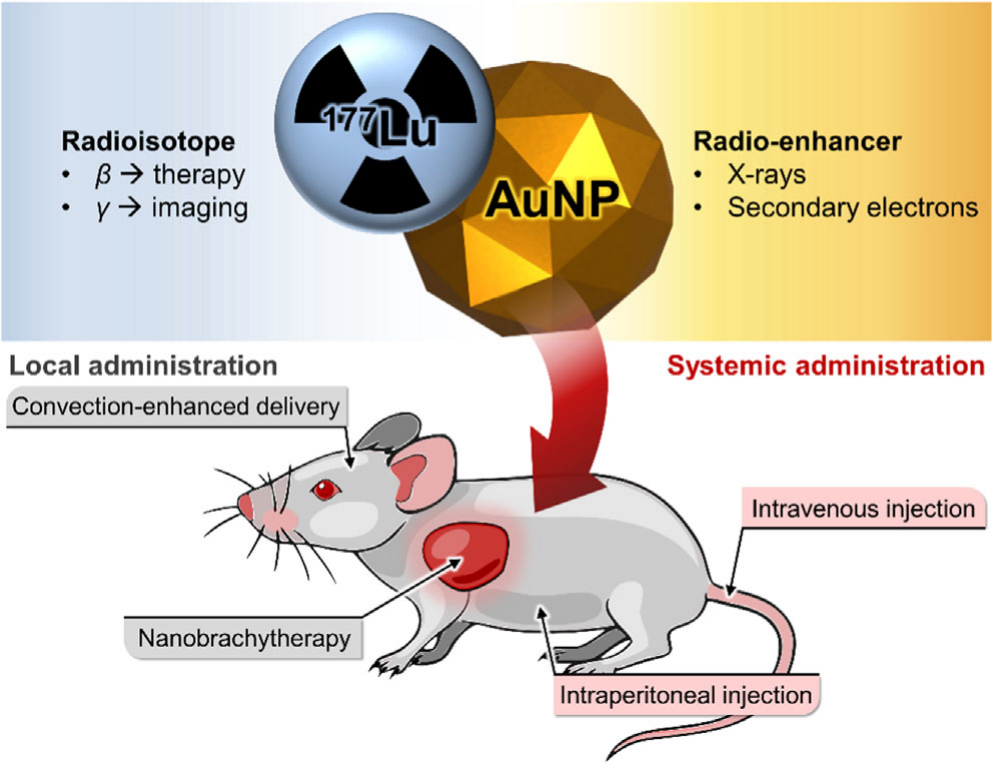
Overview of ^177^Lu‐gold nanohybrids in radiotherapeutic approaches against cancer.

Lutetium‐177 (^177^Lu) is one of the commonly used radionuclides for internal radiotherapy because of its convenient half‐life and the energy of the emitted electrons and photons (β^−^ and γ emitter).^[^ ^5^ ^]^ Some ^177^Lu‐based radiopharmaceuticals have already reached the market,^[^ ^6^ ^]^ while others are currently undergoing clinical trials.^[^ ^7^ ^]^ This article reviews the literature on nanohybrid systems incorporating ^177^Lu together with gold nanoparticles as “enhancers”, applied to internal radionuclide therapy against cancer (**Figure** **1**). A brief presentation of the lutetium‐177 radionuclide, the radioenhancing effect provided by gold nanoparticles, and tumor delivery strategies will be given before covering the state of the literature on the design and use of ^177^Lu‐gold nanohybrids in cancer therapy.

## 2 Lutetium‐177

**Figure 2:**
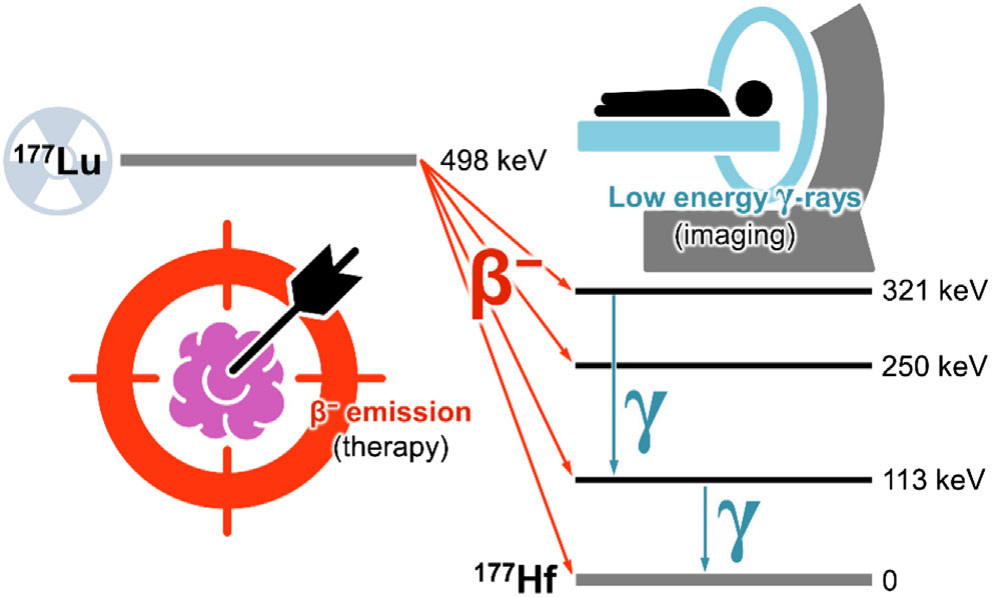
Simplified decay diagram of lutetium‐177 and potential applications of the associated radiations.

Lutetium is the heaviest element in the lanthanide series. It is naturally present on Earth in two isotopic forms, i.e., ^175^Lu and ^176^Lu. While ^175^Lu is a stable isotope accounting for most of the natural abundance, ^176^Lu is a minor and long‐lived radionuclide with a half‐life of 3.78 × 10^10^ years. Among other radioisotopes, ^177^Lu is of particular interest for therapeutic and diagnostic applications.^[^ ^8^ ^]^^177^Lu can be produced using a cyclotron, but the most practical and cost‐effective method is neutron irradiation in a nuclear reactor, either by direct activation of enriched ^176^Lu or by indirect activation of ^176^Yb → ^177^Yb, followed by decay to ^177^Lu.^[^ ^9^ ^]^ Lutetium‐177 is both a β^**−**^ and γ emitter, and this dual emission makes it suitable for not only therapy but also imaging (**Figure** **2**). ^177^Lu decays to stable hafnium‐177 with a half‐life of 6.65 days, emitting mainly β^**−**^ particles with a maximum energy of 498 keV (130 keV average energy) and γ‐radiations of 208 and 113 keV.

**Figure 3:**
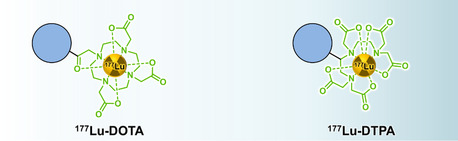
Examples of lutetium‐177 complexes with DOTA and DTPA.

The typical penetration range of the β^−^ particles emitted by ^177^Lu is *≈*2 mm, and 0.7 mm in soft tissue which is suitable in the context of radiotherapeutic approaches for energy deposition in confined volumes (e.g., tumor), while limiting radiations delivered to surrounding healthy tissues. On the other hand, γ‐photons are suitable for single‐photon emission computed tomography (SPECT) imaging. The possibility of recording scintigraphic images enables the monitoring of the in vivo behavior of ^177^Lu‐labeled radiopharmaceuticals, thus offering a theranostic (therapeutic + diagnostic) opportunity. The attachment of lutetium, in its most stable +3‐oxidation state, to a carrier system can be achieved through complexation with chelating ligands attached to the carrier. For example, macrocyclic DOTA (dodecane tetraacetic acid) or linear DTPA (diethylenetriaminepentaacetic acid) and related compounds can form fairly stable complexes with lutetium (**Figure** **3**).^[^ ^10^ ^]^

## 3 Radioenhancement by Gold Nanoparticles

In conventional radiotherapeutic approaches, the source of irradiation can be directly ionizing (ions, protons, electrons) to the cells, or indirectly ionizing (photons, neutrons) by transfer of energy to the medium, followed by the generation of free radical species. By combining radiotherapy with high‐atomic number (*Z*) metal nanoparticles, it is possible to boost the effect of irradiation by a radioenhancement phenomenon.^[^ ^11^ ^]^ In fact, high‐*Z* particles can absorb incoming radiations and generate additional species that will locally amplify the deposited dose. The processes behind the radioenhancement effect by gold are complex and can be divided into three main stages: 1) physical, with the activation and ionization of gold; 2) chemical, with the production of new chemical entities mostly from water and molecular oxygen; and finally 3) biological, with directly or indirectly induced damages. Gold nanoparticles distributed in tumor tissues widen the therapeutic window and can help overcoming some of the limitations associated with conventional radiotherapy, such as tumor radioresistance and damage to healthy tissues. Pioneering work in this field dates back to 2004, when mice with mammary carcinomas were treated with gold nanoparticles (intravenously injected) combined with X‐rays. The survival rate of the mice at 1 year was 86%, compared with 20% with X‐rays alone.^[^ ^12^ ^]^ The mechanisms underlying the radioenhancement effect^[^ ^13^ ^]^ are different whether the source of irradiation is neutral or charged. In the case of internal radiotherapeutic approaches, involving ^177^Lu‐gold nanohybrids, the low‐energy electrons and photons delivered from lutetium can interact either directly with the cellular components, the surrounding medium, or with the metal to produce additional altering species. Radioenhancement is mainly attributed to the photoelectric effect, which is the predominant mechanism by which low‐energy photons interact with gold.^[^ ^14^ ^]^ The interaction results in the release of photoelectrons from gold atoms, X‐rays, along with the generation of multiple Auger electrons.^[^ ^15^ ^]^ The range of these secondary electrons is short and the energy is deposited locally.

## 4 Administration Modes, Passive versus Active Targeting

^177^Lu‐gold nanohybrids can be administered either by direct intratumoral injection, which can be considered as a form of brachytherapy (nanobrachytherapy), convection‐enhanced delivery, or the systemic route. In the latter approach, provided they are properly coated with biocompatibilizing groups to avoid scavenging by reticuloendothelial macrophages, and if they circulate long enough in the bloodstream after intravenous injection, gold‐based hybrids can be used to deliver their radioactive payload to the tumor area by either passive or active targeting.^[^ ^16^ ^]^

Passive targeting is mostly mediated by the enhanced permeability and retention (EPR) effect, which relies on the peculiar physiology of tumor tissues that allows diffusion of nanoparticles out of the blood vessels irrigating the affected area.^[^ ^17^ ^]^ In fact, these neovessels have a fenestrated endothelium because of their inflammatory nature, making them more permeable, compared to normal blood vessels. As a result, circulating nanoparticles can diffuse from the bloodstream to the adjacent tumor tissues (extravasation). In addition, there is a lack of lymphatic drainage in solid tumors, which results in local retention and accumulation of the extravasated nanoparticles, as they cannot be cleared from the cancerous tissue.

On the other hand, active targeting relies on the functionalization of nanoparticulate systems with ligands capable of interacting with receptors overexpressed in the tumor environment, but only present at low levels in normal tissues. The grafting of ligands allows for specific molecular interactions with cells overexpressing the corresponding receptors. Upon binding, the ligand‐functionalized nanoparticles are usually internalized together with the receptor and translocate through the cell membrane via endosomal vesicles to deliver the embarked payload intracellularly. This results in increased specific uptake and improved therapeutic efficacy. The presence of targeting groups does not always ensure increased tumor accumulation of the nanoparticles, which is mainly driven by their physicochemical properties. Yet, targeting groups are likely to enhance the uptake and internalization in specific cells.

## 5 ^177^Lu‐Gold Nanoparticles‐Based Approaches

Gold nanoparticles classically exhibit minimal intrinsic toxicity and have been thoroughly investigated as platforms for internal radiotherapy approaches involving ^177^Lu. Their radiosensitizing properties make them attractive for radiotherapy, generating energetic secondary electrons that locally increase the radiation dose in the tumor, while sparing healthy tissue.^[^ ^18^ ^]^

**Figure 4:**
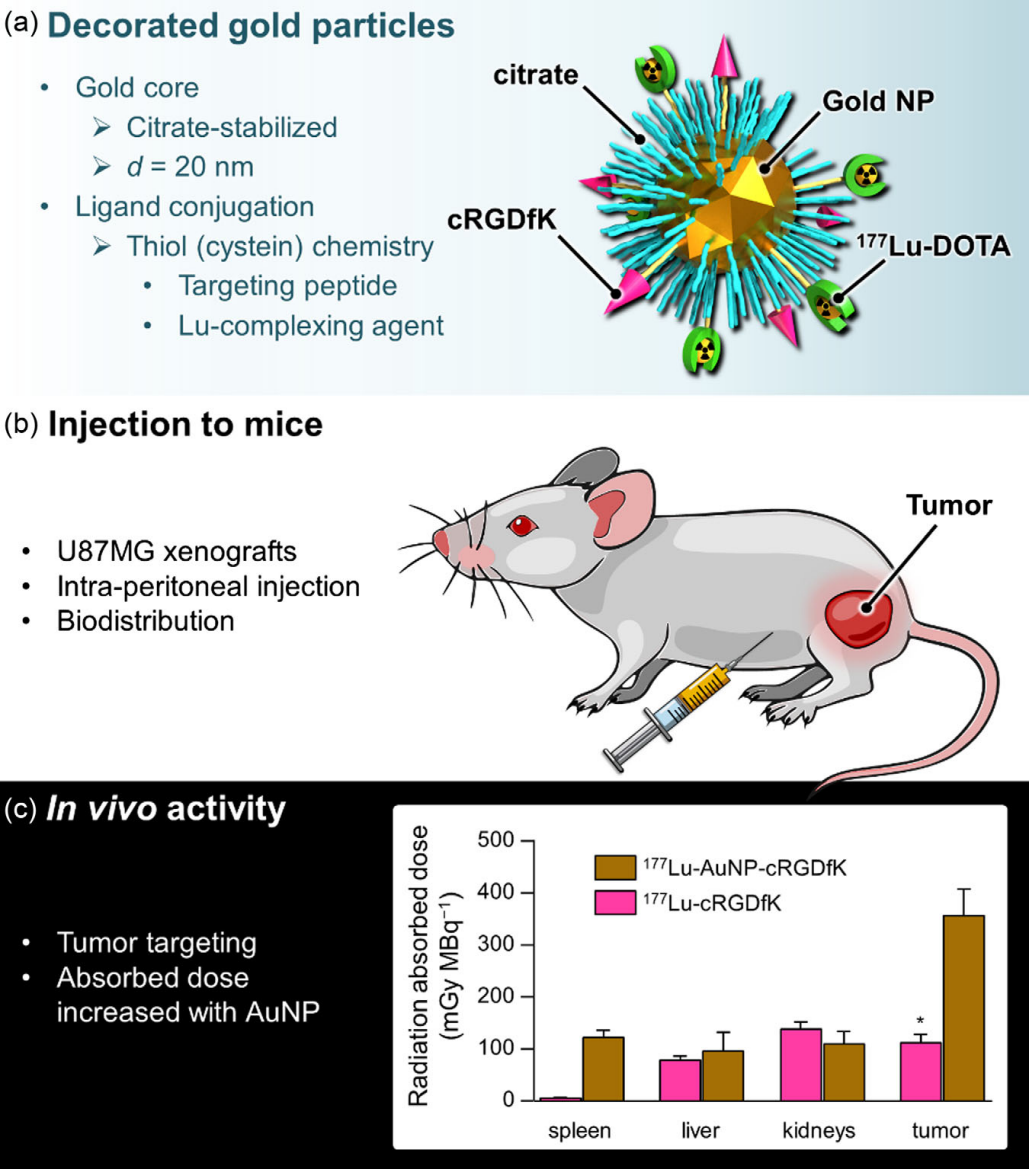
RGD‐decorated ^177^Lu‐gold nanoparticles developed by Ferro‐Flores et al.^[^ ^19^ ^]^ a) Schematic representation of the particles, b) Key parameters of in vivo studies, and c) Radiation‐absorbed doses in the main organs of interest highlighting the radioenhancement effect of gold.

To the best of our knowledge, the first use of a ^177^Lu‐gold nanohybrid in a radiotherapeutic approach against cancer was reported in 2012 by the group of Ferro‐Flores who investigated a multimeric platform based on ^177^Lu‐labeled AuNP conjugated to targeting cyclic peptides of the RGD family (**Figure** **4**).^[^ ^19^ ^]^ The latter are capable of interacting selectively with α(v)β(3) integrins that are overexpressed in numerous carcinomas. The surface of gold nanoparticles was reacted with thiol groups from cysteine‐terminated RGD peptides (for targeting) as well as cysteine‐DOTA (for lutetium chelation). The ^177^Lu‐AuNP‐RGD nanohybrid was administered by intraperitoneal injection to mice bearing U87MG tumors and was shown to target, to some extent, the affected area. The radioenhancement effect was not studied per se but the radiation‐absorbed dose (i.e., the amount of energy deposited) to the tumor was 0.35 Gy per MBq of injected activity.

Following this pioneering work, other groups investigated their own strategies for designing nanohybrids with potentiated therapeutic effects. The corresponding works are detailed in the remainder of this article and are organized according to the administration mode of the nanohybrids.

### 5.1 Local Administration

#### 5.1.1 Nanobrachytherapy

The relatively short range of ^177^Lu irradiation precludes the use of conventional brachytherapy, in which radionuclides are inserted into a sealed source positioned at the vicinity of the lesion to be treated. Instead, nanobrachytherapy approaches have been devised with direct intratumoral injections of suspensions of the nanoconstructs.^[^ ^20^ ^]^ Intratumoral injection increases the therapeutic index and solves the tumor targeting issue, as intravenously administered nanoparticles classically tend to accumulate in the liver and spleen. In addition, the nanometric size of the platforms should allow for local diffusion after administration, thus homogenizing the radiation dose deposition in the tumor.

The above ^177^Lu‐AuNP‐RGD nanoconstructs from Ferro‐Flores’ group were also administered to mice bearing integrin‐positive C6 gliomas by intratumoral injection.^[^ ^21^ ^]^^177^Lu‐AuNP‐RGD significantly inhibited C6 cell proliferation in vitro, compared to ^177^Lu‐AuNP and ^177^Lu‐RGD. In vivo, the tumor retention of ^177^Lu‐AuNP‐RGD at 96 h was *≈*35% of the injected dose (ID) and the mean tumor residence time was 61 h, which led to tumor growth inhibition. After 23 days, ^177^Lu‐AuNP‐RGD treated tumors were 27 times smaller than in the control group (untreated), 12 times smaller than ^177^Lu‐RGD, and 3 times smaller than ^177^Lu‐AuNP‐treated mice. ^177^Lu‐AuNP‐RGD was also tested for MCF7 breast cancer cell‐killing properties in vitro under laser irradiation.^[^ ^22^ ^]^ In fact, gold nanoparticles have plasmonic properties capable of converting light (mostly near‐infrared, NIR) into heat. This phenomenon generates a localized temperature increase, a process known as photothermal therapy (PTT). Laser irradiation (6 min) of preincubated ^177^Lu‐AuNP‐RGD showed strong cytotoxic effects (cell viability < 10%), as did the ^177^Lu‐generated irradiation, which was investigated in a separate set of experiments, but the PTT/radiotherapy synergy was not studied as such.

With the idea of inducing concomitant interaction with different cell receptors, the group of Ferro‐Flores similarly assembled a heteromultivalent platform made of ^177^Lu‐AuNP grafted with RGD and an aptamer, which allowed to target α(v)β(3) integrins and the vascular endothelial growth factor (VEGF), respectively.^[^ ^23^ ^]^ The construct also incorporated a nuclear localization peptide sequence to promote internalization in cancer cells. In addition to the radiotherapeutic effect, the platform was activated by laser light, to trigger a synergistic photothermal effect. Comparative in vivo assessment was conducted with the full nanohybrid (i.e., ^177^Lu‐AuNP incorporating RGD, aptamer, and nuclear localization peptide) under radiotherapy or radiotherapy + PTT conditions, and the same nanoconstruct but without ^177^Lu, under PTT conditions only.^[^ ^24^ ^]^ Hybrids were intratumorally administered four times over 21 days (2 MBq ^177^Lu activity/injection corresponding to 5.6 × 10^12^ AuNP), and retention in the tumor was monitored by Cerenkov imaging at 96 h. Tumor sizes were compared after exposure to radioactivity and/or PTT. As anticipated, the best effect was observed with the full construct with tumor sizes 126 times smaller than that of untreated mice, 28 times smaller than mice treated with thermal therapy, and 12 times smaller than the radiotherapy group.

The same research group later investigated multifunctional ^177^Lu‐AuNP grafted with both bombesin, a ligand targeting gastrin‐releasing peptide (GRP) receptor overexpressed in breast and prostate cancers, and HIV Tat(49‐57), a cell‐penetrating peptide promoting internalization and routing to the cell nucleus.^[^ ^25^ ^]^ For improved efficacy, the nanohybrid was also conjugated to ^99m^Tc, although ^99m^Tc is not a conventional radionuclide for internal radiotherapy. The ^177^Lu/^99m^Tc‐nanohybrid was evaluated in GRP receptor‐positive tumors in mice and radiation‐absorbed doses were 7.9 Gy/MBq (^177^Lu) and 0.07 Gy/MBq (^99m^Tc). The potency of the nanohybrid incorporating Tat and bombesin peptides was further evaluated in vitro for photothermal therapy.^[^ ^26^ ^]^ Laser irradiation of the cell culture induced an increase of the temperature to 46 °C, which in turn, drastically decreased cell viability down to *≈*1%. However, the synergistic effect between radiotherapy and photothermal therapy was not investigated. The two ^177^Lu‐AuNP‐based approaches involving RGD and bombesin/Tat(49‐57) peptides by the Ferro‐Flores group were reviewed by them in a 2015 article.^[^ ^27^ ^]^

**Figure 5:**
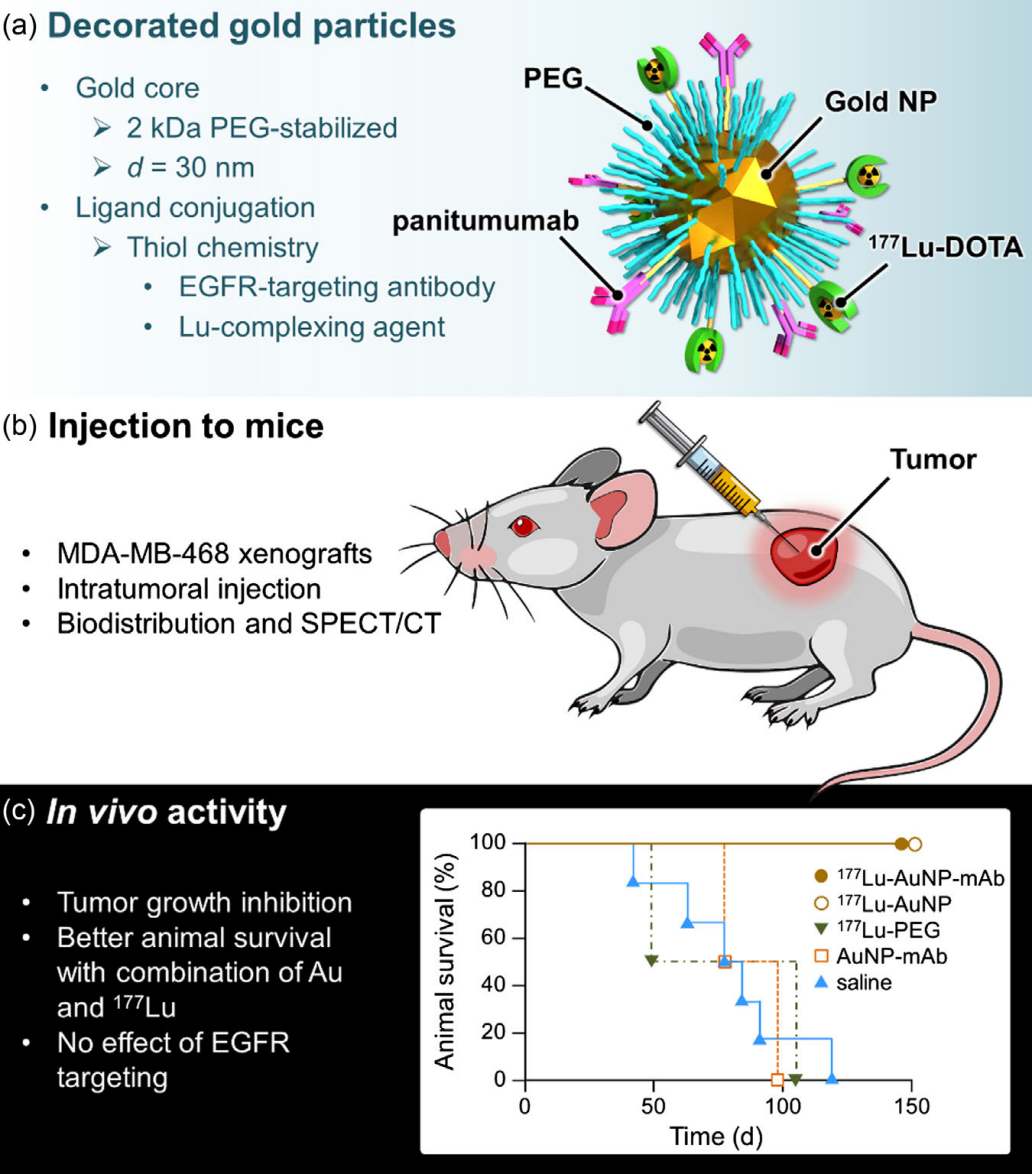
Panitumumab‐decorated ^177^Lu‐gold nanoparticles developed by Reilly et al.^[^ ^28^ ^]^ a) Schematic representation of the particles, b) Key parameters of in vivo studies, and c) Kaplan–Meier plot of animal survival after various treatments.

^177^Lu‐AuNP nanohybrids were explored in parallel by Reilly and coworkers as neoadjuvant treatment for radiotherapy of locally advanced breast cancer.^[^ ^28^ ^]^ Neoadjuvant brachytherapy involves brachytherapy as the primary intervention, followed by locoregional treatment tailored to the size of residual tumors. The AuNPs were modified with polyethyleneglycol (PEG) chains linked to DOTA, for ^177^Lu complexation, and to the monoclonal antibody panitumumab, for epidermal growth factor receptor (EGFR) targeting (**Figure** **5**). Of note, surface coating with a saturating amount of simple 2 kDa‐PEG units was required to stabilize gold nanoparticles and prevent their aggregation. The targeted ^177^Lu‐AuNP‐panitumumab hybrids were injected intratumorally to mice bearing subcutaneous EGFR‐positive MDA‐MB‐468 human breast cancer tumors. Biodistribution and single photon emission computed tomography/computed tomography (SPECT/CT) imaging in small animals were performed to assess tumor/normal organ localization, and the performance of ^177^Lu‐AuNP‐panitumumab was compared with that of the nontargeted ^177^Lu‐AuNP (without panitumumab). Radioactivity in the tumor was high 1 h after injection but decreased 2‐ to 3‐fold after 48 h. The normal organ uptake was low except for the liver and spleen, with no signs of toxicity (local doses < 1 Gy). Treatment with 4.5 MBq of radioactivity (corresponding to 6 × 10^11^ gold nanoparticles) arrested tumor growth over 90 days and survival could be prolonged up to 120 days with radiation‐absorbed doses at the tumor site in the 20–30 Gy range. Previous in vitro studies from the same group demonstrated that targeted nanohybrids exhibited high EGFR‐binding and internalization into human breast cancer cells, and were strongly cytotoxic to cells with high EGFR expression, but were less effective with breast cancer cells with low EGFR density.^[^ ^29^ ^]^ However, in vivo EGFR targeting did not provide greater tumor retention or better tumor radiation‐absorbed doses, as the two hybrids (i.e., ^177^Lu‐AuNP‐panitumumab and ^177^Lu‐AuNP) were equally effective for local treatment of breast cancer xenografts in mice. Yet, given the efficacy of the nontargeted approach, it could be used to treat locally advanced breast cancers of various phenotypes (not limited to EGFR‐positive), questioning the value of the targeted system, as nontargeted probes are easier to implement in clinics.^[^ ^30^ ^]^

Gold‐lutetium‐177 nanoparticles were likewise considered by Reilly's group as an alternative neoadjuvant regimen to treat human epidermal growth factor receptor‐2 (HER2) positive breast cancer.^[^ ^31^ ^]^ Trastuzumab, a therapeutic monoclonal antibody targeting HER2, was associated, as above, in a hybrid nanoconstruct together with ^177^Lu and gold nanoparticles. ^177^Lu‐AuNP‐trastuzumab interacted selectively in vitro with HER2‐positive cells and was ≈2.5 times more effective than ^177^Lu‐AuNP in decreasing clonogenic survival and inducing DNA double‐strand breaks in MDA‐MB‐361 cells after exposure to 20 MBq mg^−1^ Au. The ability of ^177^Lu‐AuNP‐trastuzumab to control tumor growth in vivo by intratumoral injection was evaluated in mice bearing tumor xenografts (MDA‐MB‐361) and its efficacy was compared to that of ^177^Lu‐AuNP without trastuzumab. Mice were treated by a single intratumoral injection of 3 MBq of the nanohybrids (corresponding to 0.15 mg AuNP, 5.5 × 10^11^ particles) and were monitored over 16 days. The tumor growth index of ^177^Lu‐AuNP‐trastuzumab was 1.7‐ and 2.2‐fold significantly lower compared to ^177^Lu‐AuNP or untreated mice, respectively. In contrast to previous works on panitumumab nanohybrids developed for the treatment of EGFR‐positive cancers (*vide supra*), tumor growth arrest could not be achieved here with the trastuzumab nanohybrids. Yet, the presence of the targeting antibody appears to improve the therapeutic index with a superior in vivo activity of the antibody‐conjugated system compared to simple ^177^Lu‐AuNP.

Based on the observation that resistance to HER2‐targeted therapies in breast cancer is sometimes associated with an increased expression of epidermal growth factor receptors (EGFR), Reilly's group developed a radiation nanomedicine targeting the two receptors that was evaluated in vitro.^[^ ^32^ ^]^^177^Lu‐Au nanoparticles were grafted both with trastuzumab (to target HER2) and panitumumab (to target EGFR), and the nanohybrids were studied in interaction with breast cancer cells overexpressing either HER2, EGFR, or both receptors. Single‐antibody‐functionalized ^177^Lu‐AuNP interacted only with cells expressing the corresponding receptor (HER2 or EGFR), whereas dual‐antibody‐functionalized ^177^Lu‐AuNP interacted with cancer cells expressing HER2, EGFR, and both receptors. The ^177^Lu‐AuNP‐trastuzumab‐panitumumab hybrid was more potent in selectively depositing high‐absorbed doses in the nucleus of HER2‐EGFR cells, compared to the two single‐receptor‐targeted‐hybrids (i.e., ^177^Lu‐gold‐trastuzumab and ^177^Lu‐gold‐panitumumab) and nontargeted ^177^Lu‐AuNP.

**Figure 6:**
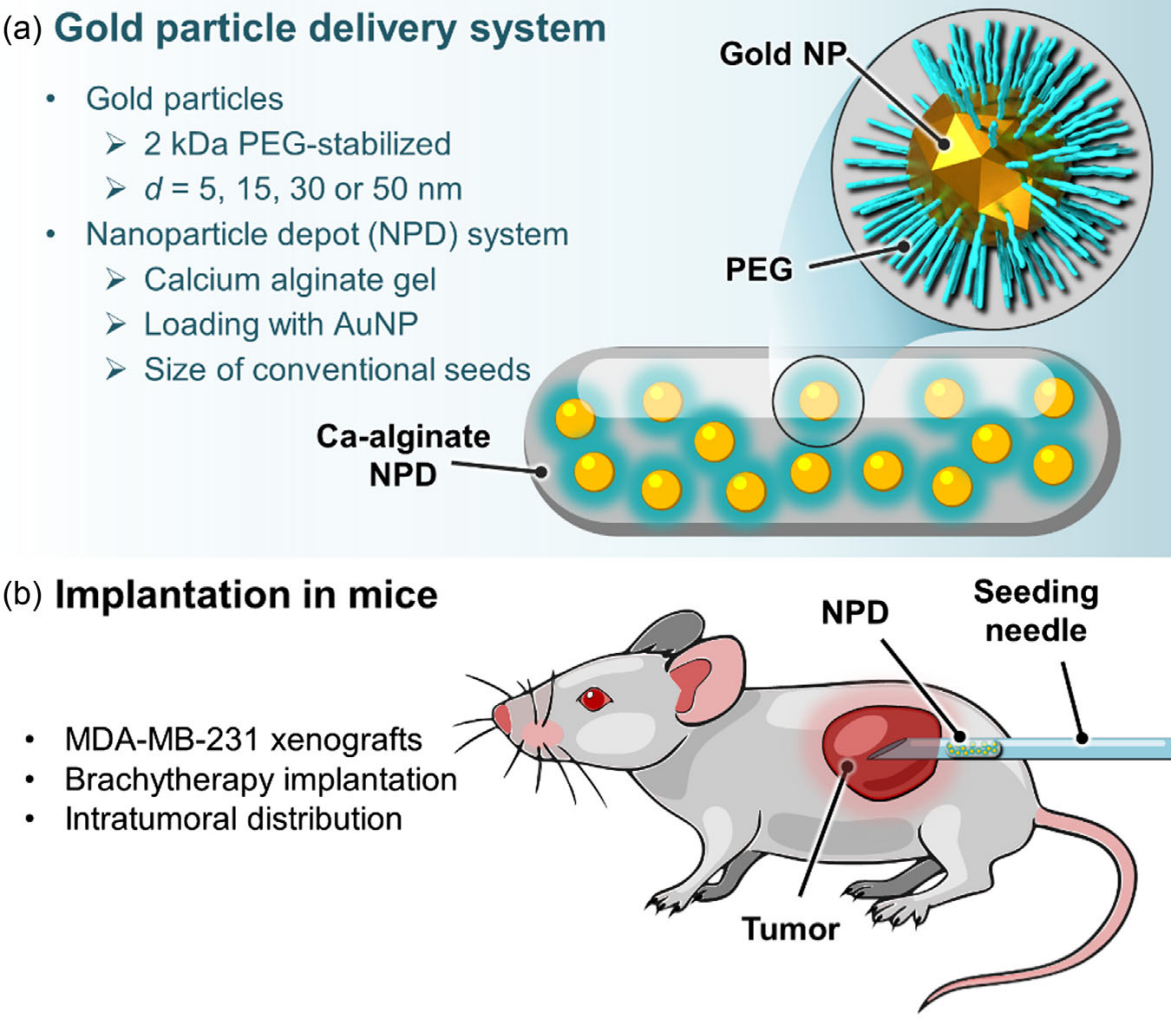
Alginate‐based nanoparticle depot system developed by Reilly et al.^[^ ^33^ ^]^ a) Schematic representation of the AuNP delivery system and b) Illustration of the implantation of the NPD in tumor‐bearing mice.

One obstacle to advancing intratumoral delivery is the difficulty of precisely positioning AuNP in tumors to achieve predictable homogenous radioactivity distribution and dose deposition. Because of the size of the tumors, treatment of human patients would require numerous and spatially distributed injections (with high accuracy to minimize heterogeneities). In an attempt to solve this problem, Reilly's group has developed a nanoparticle depot (NPD) system consisting of a porous alginate reservoir for AuNPs, allowing controlled local release and diffusion (**Figure** **6**).^[^ ^33^ ^]^ NPD has the same dimensions (a few millimeters in length) as the brachytherapy seeds commonly used in patients for local tumor irradiation, so they can be precisely positioned using the technique used for implanting permanent seeds. The release and diffusion of the AuNP from NPD were studied in a phantom and in a mouse model after xenograft implantation. In both cases, a sustained release of AuNP was observed. Monte Carlo simulations indicated that radioactivity remained confined to the implant site and dose distributions were predictable and concentric, unlike intratumoral injection of AuNP, which produced irregular and variable dose distributions over time.^[^ ^34^ ^]^ Yet, in vivo experiments with the NPD system showed a heterogeneous intratumoral distribution of AuNP, in contrast to what was observed in the phantom model. This result highlights the complexity of living systems and the somewhat limited relevance of simple models.

Monte Carlo simulation was also used by the Ferro‐Flores group to investigate how fibrosis in tumor tissue reduces absorbed radiation doses.^[^ ^35^ ^]^ Gold nanoparticles were labeled with ^177^Lu and octreotide peptide (OCT) capable of binding to somatostatin receptors. ^177^Lu‐AuNP‐OCT retention by HeLa cells was studied in fibrotic and nonfibrotic microenvironments. The residence time of ^177^Lu‐AuNP‐OCT was found to be higher in the fibrotic tissue (*≈*22 h) than in the nonfibrotic tissue (*≈*17 h). Simulation results showed only a small increase (*≈*6%) in the average energy deposited in a fibrotic model, compared with a nonfibrotic one and the increase in the absorbed radiation dose reached 33% at a depth of 10 mm.

**Figure 7:**
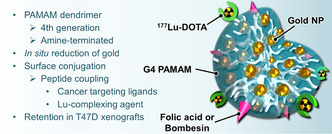
Schematic representation of the dendrimeric system developed by Ferro‐Flores et al.^[^ ^36^ ^]^ for the targeted delivery of AuNP and ^177^Lu.

In an approach that differs from those classically involving gold nanoparticles as central platforms, the same group assembled ^177^Lu‐labeled dendrimeric cores encapsulating gold nanoparticles. Folate and bombesin molecules were conjugated at the surface of the dendrimers for simultaneous targeting of folate receptors and gastrin‐releasing peptide receptors (overexpressed in breast cancer cells) (**Figure** **7**).^[^ ^36^ ^]^ In vitro studies indicated selective uptake of ^177^Lu‐dendrimer‐AuNP‐folate‐bombesin in T47D cancer cells that was inhibited in the presence of competing free bombesin or folic acid. Intratumoral injection of the nanoconstruct to tumor‐bearing mice showed local retention up to 96 h, as evidenced by SPECT/CT imaging. These experiments were however preliminary with mention of neither therapeutic efficacy nor added value of the ligands in vivo. The dendrimer incorporating gold, folate, and bombesin was further evaluated as regards photothermal activation in vitro.^[^ ^37^ ^]^ Laser irradiation of cells incubated with dendrimer‐AuNP‐folate‐bombesin decreased the cell viability but there was no indication of the synergy between radio‐ and photothermal therapies. A few years later, related gold‐incorporating dendrimeric structures, functionalized with folate, bombesin, and ^177^Lu were investigated in vitro by Fang and coworkers for selective interactions with HEL‐299 lung cancer cells.^[^ ^38^ ^]^

Although these highly functional systems, which combine multiple ligands, appeared promising on paper for ultratargeted therapy, they quickly encountered the challenges posed by living systems and the complexity of the tumor microenvironment. This complexity has thus far prevented the validation of the ^177^Lu‐dendrimer‐AuNP‐folate‐bombesin platform in preclinical therapeutic studies.

Nanobrachytherapy offers a number of advantages in preclinical studies, such as a radiation source directly released inside of the body (decreasing required doses) and a localized irradiation zone, resulting in fewer side effects on surrounding normal tissues. On the less advantageous side, distribution of ^177^Lu‐AuNP throughout the tumor volume is to some extent heterogeneous, and nanobrachytherapy can only be applied to accessible pathological sites and not to deeply buried areas.

#### 5.1.2 Convection‐Enhanced Delivery

The administration of drugs to the central nervous system (CNS) by intravenous injection is generally hampered by the crossing of the blood–brain barrier (BBB), which regulates transfers from the circulatory system. An alternative delivery mode is convection‐enhanced delivery (CED), which consists of inserting a catheter into the brain using image guidance to deliver therapeutics directly through the interstitial spaces of the CNS via a pressure gradient.^[^ ^39^ ^]^ This technique makes it possible to bypass the BBB and perfuse deep targets close to and downstream of the infusion site.

**Figure 8:**
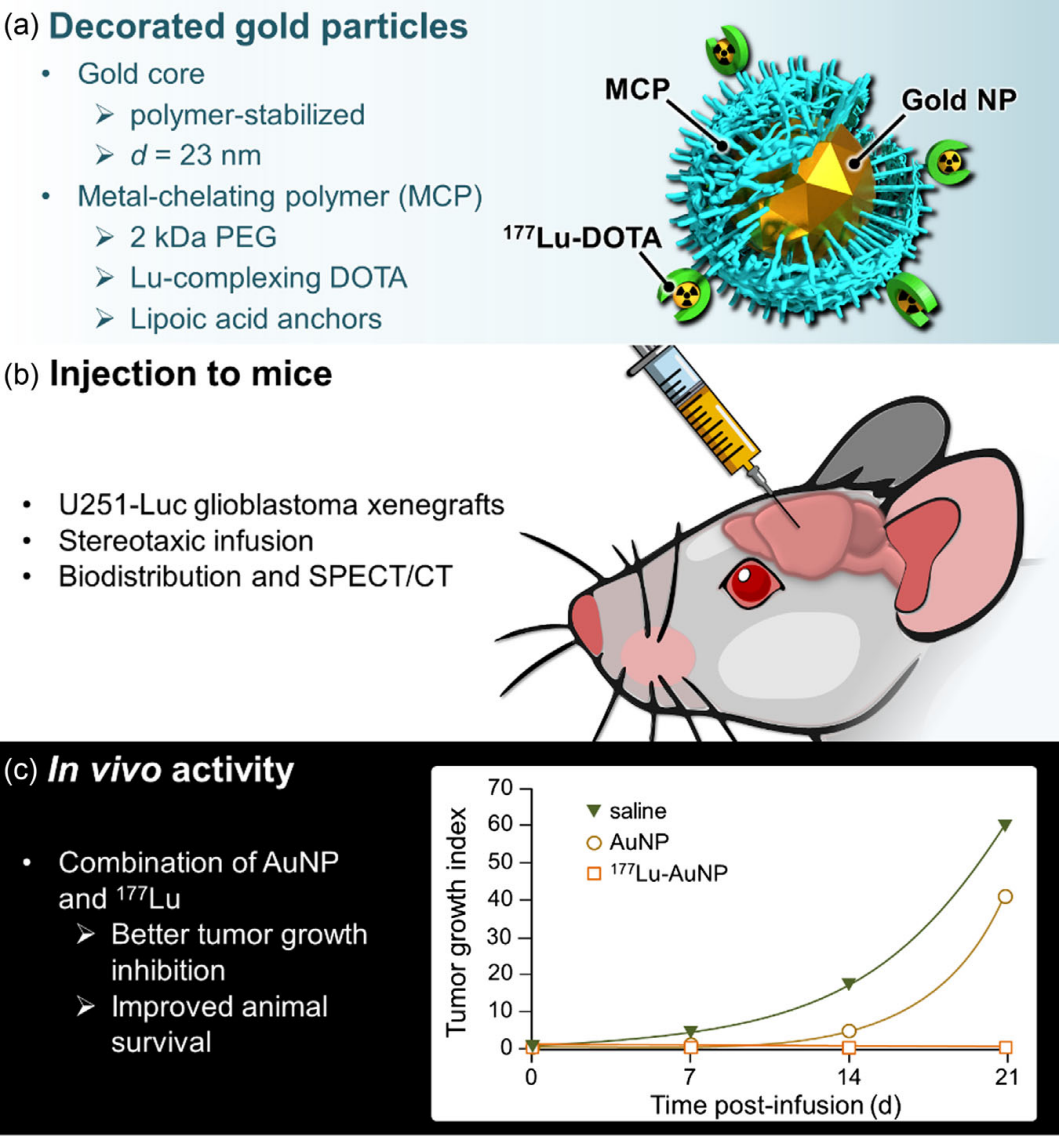
Convection‐enhanced delivery of gold nanoparticles decorated with lutetium‐complexing‐polymer developed by Reilly et al.^[^ ^40^ ^]^ a) Schematic representation of the particles, b) Illustration of the in vivo stereotactic administration, and c) Tumor growth inhibition effect highlighting the benefit of combining AuNP with ^177^Lu.

CED‐locoregional delivery of ^177^Lu‐AuNP was implemented by Reilly and coworkers and applied to glioblastoma multiforme (GBM), which is the most common and lethal primary brain tumor (**Figure** **8**).^[^ ^40^ ^]^ A chelating polymer, precomplexed to ^177^Lu, was conjugated to AuNP (*d* = 23 nm). The functional ^177^Lu‐AuNP were administered *via* CED to mice xenografted with orthotopic U251‐Luc glioblastoma cells in the right cerebral hemisphere. SPECT/CT imaging showed retention of radioactivity at the infusion site up to 21 days, whereas ^177^Lu complexed to the chelating polymer (no gold) was rapidly cleared within 2–3 days. CED of ^177^Lu‐AuNP (1 MBq, 4 × 10^11^ AuNP) strongly inhibited the growth of U251‐Luc with a spectacular tumor radiation‐absorbed dose of *≈*600 Gy. It is worth noting that doses were 93‐fold lower in the “normal” right cerebral hemisphere (where the tumor was implanted) and 2000‐fold lower in the contralateral left hemisphere, underscoring local confinement of the nanohybrids. The approach significantly prolonged the median survival of treated mice compared to control mice up to 3.8‐fold, without toxicity to healthy tissue. Magnetic resonance imaging (MRI) recorded 28 days after infusion of ^177^Lu‐AuNP revealed no evidence of residual tumor in the brain, unlike control mice treated with AuNP or normal saline.

The interaction of ^177^Lu‐AuNP hybrids in GBM was investigated at the cellular level by Silva, Paulo, and coworkers to target neurokinin‐1 receptors (NKR1), expressed on the surface of glioma cells.^[^ ^41^ ^]^ The main endogenous ligand of NK1R is substance P (SP) that belongs to the family of tachykinin peptide neurotransmitters. ^177^Lu‐AuNP were functionalized with a derivative of SP and were evaluated on the U373 cell line. Rapid and massive uptake of ^177^Lu‐AuNP‐SP in U373 cells was observed, and radiocytotoxicity evaluation indicated a marked reduction in the surviving fraction (clonogenic assay) and in cell viability (MTT assay), even at the lowest dose tested (0.3 MBq) with a superior effect to that of simple ^177^Lu‐AuNP (no SP ligand).

While efficient, nanobrachytherapy and convection‐enhanced delivery are rather invasive processes. For example, in CED approaches, a small incision is made in the skin to expose the skull before catheters are stereotactically inserted through a burr hole into the interstitial spaces of the brain using image guidance. Provided they are as potent as those administered locally, radiopharmaceuticals could be administered in a less invasive way, calling for the development of systemic delivery routes.

### 5.2 Systemic Administration

In addition to being less invasive, the systemic administration of the nanohybrids offers the possibility of reaching deep areas in the body with no a priori on the precise localization of the tumor. In fact, pharmacokinetics and targeted biodistribution are mainly driven by physicochemical properties of the nanometric platform and by the physiological environment of the tumorous tissue, respectively. This aspect is important to consider in the treatment of spreading metastatic cancers whose precise diagnosis remains problematic in the early stages of the disease.

**Figure 9:**
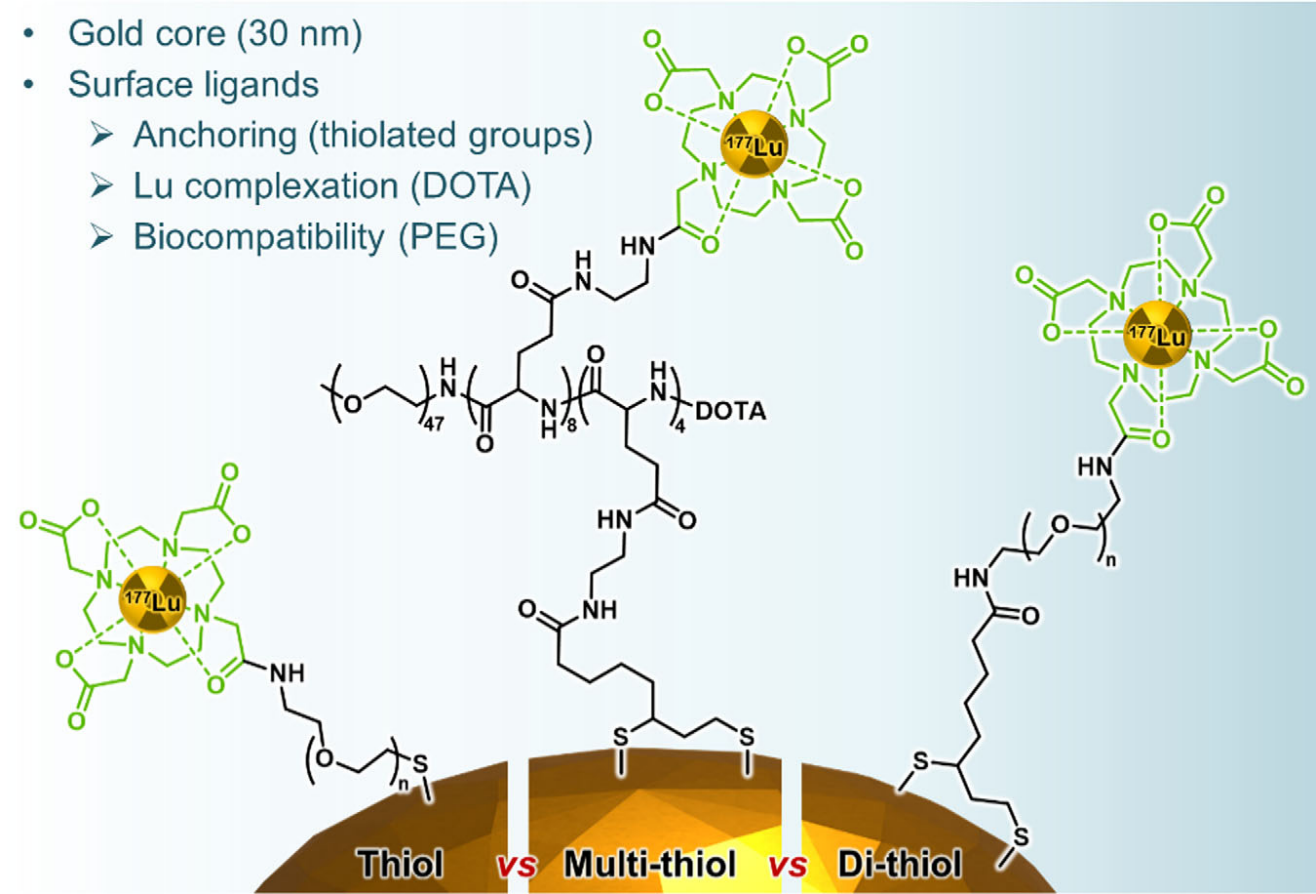
Different ^177^Lu‐DOTA‐PEG‐complexing ligand types studied by Reilly et al.^[^ ^42^ ^]^

One of the key issues with systemic approaches is the colloidal stability of the gold‐based carriers, which can be provided by biocompatibilizing groups (e.g., polyethyleneglycol (PEG)) attached at the surface of the nanoparticles. In vivo, these elements confer “stealth” properties and minimize uptake by the reticuloendothelial system, thus allowing extended circulation times. PEG chains and their derivatives are classically bonded to gold via thiol units. The group of Reilly investigated the benefit of adding multiple S‐Au bonds per ligand for the overall stabilization of PEG‐coated ^177^Lu‐gold nanohybrids (**Figure** **9**).^[^ ^42^ ^]^ Gold nanoparticles were decorated with PEG chains functionalized at one end with DOTA (for ^177^Lu complexation), and at the other end with either single, double or multiple thiols. The stability of the three nanoconstructs was assessed both in vitro (aggregation and dissociation of ^177^Lu) and in vivo by looking at elimination and excretion. It was found that multiple‐thiol ligands provided better stability and prolonged the circulation time with 5.8% ID left in the bloodstream 6 h after intravenous injection. This value compares favorably with that of double‐(2.4% ID) and monothiol ligands (1.5% ID) at the same time point. Biodistribution studies showed accumulation in the liver and spleen, and radioactivity measurements in feces and urine revealed the renal excretion of some free ^177^Lu (gold‐unbound), whereas gold nanoparticles were most likely excreted through the hepatobiliary pathway. Urinary excretion of radioactivity, 168 h after injection, was 61.4 ± 3.5% ID for mice injected with AuNPs bearing monothiol ligands, which is 1.4 and 1.6 times significantly higher than for AuNPs coated with di‐ and multithiol ligands, respectively. These results highlight the benefits of multiple‐thiol chemistry in stabilizing PEG grafting onto AuNPs and the construction of ^177^Lu‐AuNPs for in vivo radiation treatment of cancer.

#### 5.2.1 Passive Targeting

Intravenously injected nanometric platforms can diffuse through leaky endothelium junctions of tumor blood vessels and accumulate in solid tumors. This process is known as the enhanced permeability and retention (EPR) effect and referred to as passive targeting.

**Figure 10:**
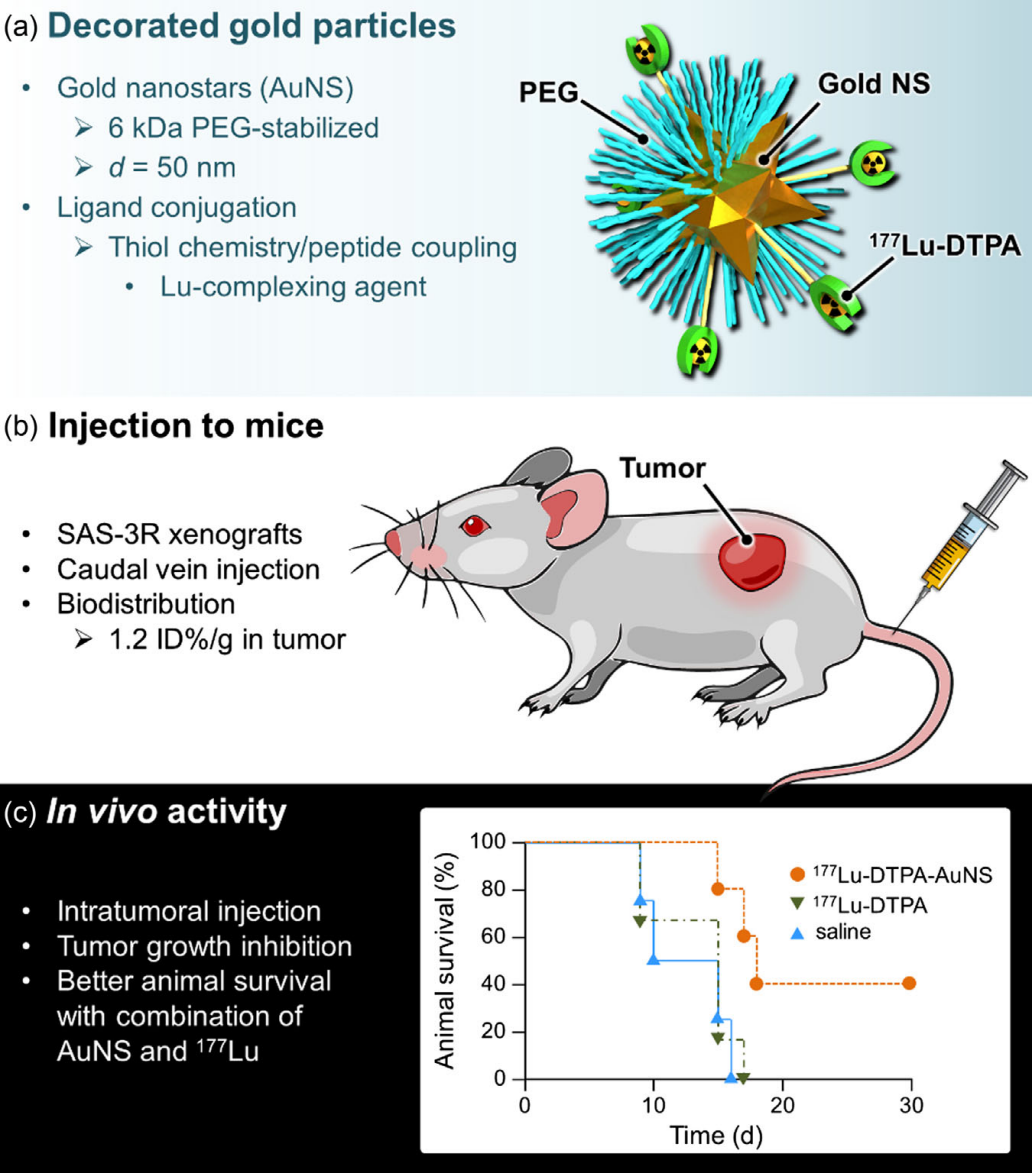
^177^Lu‐DTPA‐decorated gold nanostars developed by Wu et al.^[^ ^43^ ^]^ a) Schematic representation of the particles, b) Key parameters of the in vivo evaluation, and c) Improved animal survival highlights the benefit of combining AuNS with ^177^Lu.

Wu and coworkers used gold nanostars (AuNS) to design radiotherapeutic hybrids against head and neck squamous cell carcinoma (**Figure** **10**).^[^ ^43^ ^]^ AuNS belong to a class of nanoparticles with a specific shape consisting of a solid core decorated with multiple branches. In addition to the radioenhancement effect, AuNS also have plasmonic properties that can convert near‐infrared light (NIR) to heat for PTT applications. AuNS were functionalized with PEG using thiol‐chemistry, and ^177^Lu was associated with the PEGylated particles through complexation with DTPA, a chelating agent for lutetium, grafted at the surface of the nanoobjects. ^177^Lu‐AuNS were intravenously injected to xenografted mice and passive accumulation in the tumor (*≈*1.2% ID g^−1^) was detected after 24 h with, however, limited potency (most of the radioactivity was found in the reticuloendothelial organs, i.e., liver and spleen). In the same study, the authors also explored direct intratumoral injection of ^177^Lu‐AuNS. Quantitative analysis indicated high levels of radioactivity in tumor lesions up to 72 h after injection and only little radioactivity in normal organs. Retention of the nanohybrid in the tumor was observed by micro‐SPECT/CT imaging, and therapeutic potency was assessed by bioluminescent imaging of luciferase‐expressing cancer cells. Nearly full suppression of the luciferase activity was observed with the intratumorally injected ^177^Lu‐AuNS but not with control ^177^Lu‐DTPA (no AuNS), underscoring the key contribution of gold. Internal radiotherapy was also combined with photothermal therapy. The tumor area containing ^177^Lu‐AuNS was exposed to near‐infrared laser irradiation for 6 min. Although no complete tumor regression could be observed, the residual masses remaining after laser‐irradiation were nonviable scar tissues rather than live cancerous cells.

The aforementioned study shows the somewhat limited efficacy of passive targeting, which has led most research groups involved in systemic administration of ^177^Lu‐AuNP to explore active targeting approaches in the hope of improving selective accumulation in the tumors.

#### 5.2.2 Active Targeting

In active targeting, ligands capable of binding to specific receptors overexpressed by cancerous cells are attached to the surface of the nanometric platform, directing it toward its therapeutic target. Active targeting can increase the selectivity of delivery provided that the intravenously injected nanocarrier system can reach and diffuse into the affected tissue. In the context of internal radiotherapy, active targeting approaches are intended to maximize the local radiation‐mediated cytotoxic effect on cancer cells.

An early study on active targeting in vivo was reported by the group of Robertson who developed nanoparticle cores incorporating cold lutetium/gadolinium, together with radioactive ^177^Lu, for the targeting of pulmonary cancerous metastases.^[^ ^44^ ^]^ The nanoparticles were coated with a gold shell, providing access to [^177^Lu]Lu_0.5_Gd_0.5_(PO_4_)@Au. In contrast to other platforms in which ^177^Lu is chelated by organic molecules at the surface of gold, in the above construct the radioactive isotope is bound within the crystalline core, thus enhancing in vivo stability. Gold‐coated nanoparticles were then made biocompatible through decoration with PEG units and further conjugated to monoclonal antibodies (mAb‐201B) targeting thrombomodulin receptors in the lungs. The [^177^Lu]Lu_0.5_Gd_0.5_(PO_4_)@Au‐mAb hybrid was administered via tail vein injection to mice pretreated with clodronate liposomes, which deplete the macrophage population available to clear particles from the bloodstream. The lung uptake of mAb‐labeled nanoconjugates was found to be rapid and binding specific. At 10 min postinjection, more than 95% ID was detected in the lungs. This uptake was retained for at least 1 h, as monitored by SPECT/CT. After 24 h, the proportion in the lungs decreased, while the amount in the liver increased slightly and more significantly in the spleen, with some excretion through the hepatobiliary route. Organs were harvested for transmission electron microscopy and the ultrastructural loci of [^177^Lu]Lu_0.5_Gd_0.5_(PO_4_)@Au‐mAb were determined: most of the injected dose was localized in type I and type II pneumocytes.^[^ ^45^ ^]^ Although the system seemed promising for targeting pneumocytes, therapeutic applications were not explored further. The question therefore remains open as to whether this invention has some potential for treating metastatic cancer in the lungs.

Cetuximab is a chimeric EGFR‐targeting antibody used to treat patients with EGFR‐overexpressing cancers including metastatic colorectal cancer. Smith and coworkers examined the parameters affecting the cell‐killing efficacy of cetuximab‐functionalized ^177^Lu‐AuNP in the context of radioimmunotherapy, i.e., intravenous administration of targeted conjugates carrying cytotoxic radionuclides and seeking out primary tumors and metastases.^[^ ^46^ ^]^ These parameters included target affinity, duration of association with the target cells, heterogeneity in intratumor dose‐distribution, and tumor growth inhibition efficacy. Cetuximab and ^177^Lu were conjugated to PEG units attached to gold via S‐Au bonds. In another report, Smith's group used a combination of physicochemical techniques to study in detail the grafting efficiency of gold nanoparticles with functional PEG linkers, ^177^Lu and cetuximab.^[^ ^47^ ^]^ Their findings indicated that atomic force microscopy (AFM) imaging combined to force distance spectroscopy was a reliable method for the characterization of chemical and biological modifications of gold nanoparticles. The in vitro affinity of ^177^Lu‐AuNP‐cetuximab for EGFR was measured at 20 nM (K_Dis_). Ligand binding to the EGFR resulted in rapid clathrin‐dependent endocytosis. Survival of MDA‐MB‐468 cells treated with 5 MBq of EGFR‐targeted ^177^Lu‐AuNP‐cetuximab for 4 h led to almost complete cell death after 11 days. Biodistribution of ^177^Lu‐AuNP‐cetuximab in xenografted mice (MDA‐MB‐468 cell line) showed some uptake in the tumor 4 h after injection, but the largest uptake was in the liver and spleen. Dose distribution monitoring by storage‐phosphor autoradiography across tumor sections showed a coefficient of variation to the mean activity of *≈*15%, suggesting an inhomogeneous distribution of the nanoparticles within tumors. Of note, in vivo therapeutic potency was not investigated.

**Figure 11:**
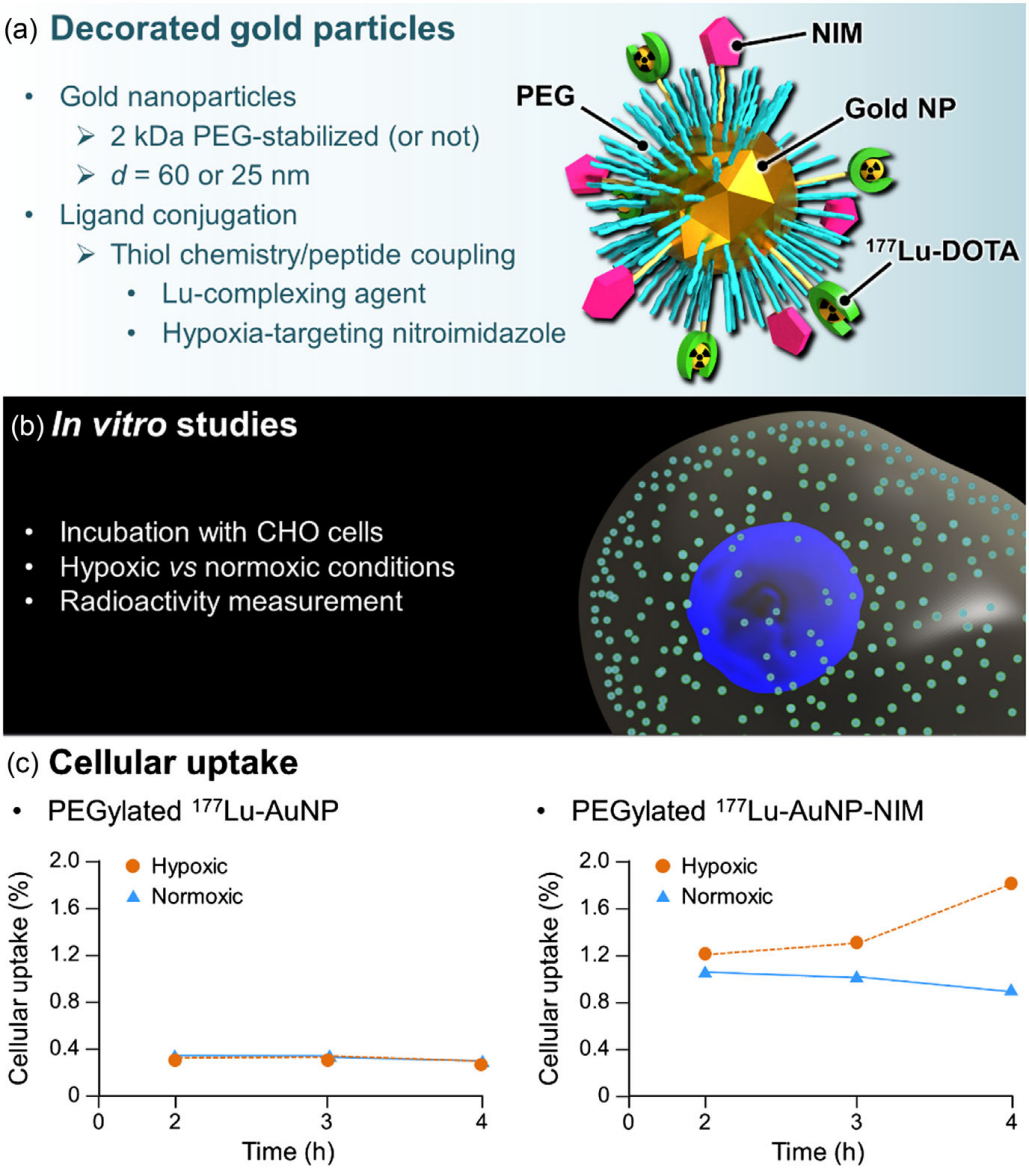
Hypoxia‐targeting ^177^Lu‐DOTA‐decorated AuNP developed by Mallia et al.^[^ ^48^, ^49^ ^]^ a) Schematic representation of the particles, b) Key parameters of in vitro studies, and c) Comparison of the in vitro hypoxia‐targeting ability of pegylated ^177^Lu‐AuNP with and without NIM ligands.

Tumor hypoxia is a pathological condition due to insufficient blood supply in rapidly growing tumors. Mallia and coworkers have developed a selective hypoxia‐targeting platform based on gold nanoparticles associated with complexed ^177^Lu, and nitroimidazole (NIM), an extensively studied bioreductive marker for noninvasive detection of hypoxia.^[^ ^48^ ^]^ Nitroimidazoles can undergo selective, oxygen‐dependent, accumulation and retention in hypoxic cells through a series of reductions by nitroreductase enzymes. Preliminary in vitro investigations conducted with ^177^Lu‐AuNP‐NIM showed improved uptake of the radiotracer under hypoxic conditions compared to normoxic conditions, with a hypoxic/normoxic ratio of 9.2, 4 h postincubation, demonstrating excellent hypoxia selectivity. Of note, ^177^Lu‐AuNP (without NIM) showed very low cellular uptake under either hypoxic or normoxic conditions. Yet, in vivo biodistribution of ^177^Lu‐AuNP‐NIM in Swiss mice bearing fibrosarcoma tumors showed very fast clearance of the nanoparticles. About 70% of the injected activity was excreted 3 h postinjection and only 0.2% ID g^−1^ was detected in the tumor area. One possible explanation for the unfavorable in vivo behavior of ^177^Lu‐AuNP‐NIM is the absence of biocompatibilizing groups at the surface of the carrier system, which would have limited its capture by the immune system. In fact, NIM and ^177^Lu‐DOTA were initially bonded to gold through a short hydrocarbon‐chain linker and S‐Au bonds. To try to solve this issue, the same group recently reported an alternative hypoxia‐targeting platform made of gold nanoparticles coated, this time, with 2 kDa‐PEG chains that were functionalized at their extremity with DOTA (for ^177^Lu complexation) and NIM.^[^ ^49^ ^]^ The PEGylated particle showed increased cellular uptake in CHO cells under hypoxic conditions, even though the effect was less spectacular than that observed with non‐PEGylated particles (**Figure** **11**). The PEGylated nanohybrid was administered by intravenous injection to Swiss mice bearing fibrosarcoma tumors. Tumor/blood and tumor/muscle ratios were high 24 h post‐injection, but rapid clearance was again observed. The authors suggested that ^177^Lu‐AuNP‐NIM were excreted, but without direct evidence, as neither urine nor feces were collected. This rapid excretion is somewhat surprising given the size of the nanoconstructs (around 28 nm), which should have accumulated transiently in the liver before being eliminated via the hepatobiliary route. This liver accumulation should have been observed at various time points, but virtually all the radioactivity had vanished from the body in less than 4 h. One hypothesis would be the lack of stability of the nanometric platform with premature release of free ^177^Lu and its elimination by the renal route. In addition, the radiotherapeutic effect could not be investigated, which raises the question of the system's potential.

#### 5.2.3 Tumor Radioimmunotherapy

In addition to improving the therapeutic outcome of local radioisotope therapy, ^177^Lu‐Au could also induce anticancer immunity and suppress the growth of distant tumors when combined with immune checkpoint inhibitors. With this objective in mind, the team of Yang investigated lutetium‐labeled gold nanoclusters (^177^Lu‐AuNC) that were prepared by simple chelation between glutathione ligands (bonded at the surface of gold) and the radionuclide.^[^ ^50^ ^]^ The nanohybrid was first evaluated in vitro and found to be potent in 4T1 cell viability tests and clonogenic assays. In vivo behavior of ^177^Lu‐AuNC was then examined by local injection in the tumor and by intravenous administration. Intratumoral administration of ^177^Lu‐AuNC to 4T1 tumor‐bearing mice, followed by SPECT/CT imaging and ex vivo biodistribution studies, evidenced good retention of the nanohybrid in the tumor site compared to free ^177^Lu. Furthermore, intratumorally injected ^177^Lu‐AuNC nearly suppressed tumor growth at a dose of 2.7 MBq, whereas systemic administration of a higher dose of the same nanohybrid (7.4 MBq) only partially inhibited the growth by *≈*75%. Based on the observation that ablative treatments such as radiotherapy can induce tumor‐specific immune responses, the authors focused on the maturation of dendritic cells, which are capable of capturing and processing antigens to activate T cells and trigger subsequent immune responses. Intratumoral injection of ^177^Lu‐AuNC was found to induce about 35% maturation of dendritic cells 3 days after treatment, suggesting an immunostimulant effect. The immunoregulation capability of ^177^Lu‐AuNC was also tested in a distant tumor model. Tumors were implanted on the two sides of the back of the mice. While one of the tumors was treated intratumorally with ^177^Lu‐AuNC, the distant one was harvested for analysis. Unexpectedly, some increase in PD‐L1 expression was detected in the distant tumor, prompting the authors to combine internal radioisotope therapy with immunotherapy to inhibit the remote tumor growth through coadministration of an immune checkpoint inhibitor (αPD‐L1). Treatment of the primary tumor with ^177^Lu‐AuNC combined with repeated injections of αPD‐L1 showed marked inhibition of distant tumor growth with an increase of cytotoxic T lymphocytes. The treatment effectively prolonged the lifespan of the mice by a factor of two, compared with controls. These results suggest that the immune memory effect induced by the ^177^Lu‐AuNC/αPD‐L1 treatment could prevent tumor cell recurrence.

Although targeted and nontargeted systemic administrations are less invasive, a key current limitation is the interaction of the nanohybrids with the reticuloendothelial system, resulting in uptake by macrophages and temporary accumulation in the liver and spleen. This undesirable accumulation can lead to side toxicity resulting from exposure of the healthy organs to ^177^Lu irradiation. ^177^Lu‐gold nanohybrid chemistry therefore needs to be optimized to avoid scavenging by macrophages and favor the more straightforward urinary excretion.

## 6 Other Radiolabeled Gold Nanoparticle Systems

In addition to ^177^Lu, other radionuclides have been combined with gold nanoparticles to potentiate internal radiotherapeutic effects. These include Astatin‐211 (^211^At, α emitter, *t*_1/2_ = 7.2 h), Actinium‐225 (^225^Ac, α, 9.9 d), Gold‐198 (^198^Au, β^−^, 2.9 d), Iodine‐131 (^131^I, β^−^/γ, 8 d), Iodine‐125 (^125^I, ε, 59.4 d), Indium‐111 (^111^In, ε, 67.9 h), and Palladium‐103 (^103^Pd, ε, 16.9 d). **Table** **1** gives an overview of the different nanoconstructs that have been preclinically evaluated against different cancer types.

**Table 1:** Overview of radiolabeled (other than ^177^Lu)‐gold hybrids and their therapeutic uses.

Isotope	Nanoconstruct	Therapeutic use
^211^At	^211^At‐AuNP‐Trastuzumab^[^ ^53^ ^]^	In vitro, target HER2 receptors, evaluated on SKOV‐3 ovarian cancer cells with higher affinity and cytotoxicity than unmodified nanoparticles.
	^211^At‐AuNP‐Substance P^[^ ^54^ ^]^	In vitro, target NK1 receptors, evaluated on human gliomas T98G cells with reduction of metabolic activity in a dose‐dependent manner with ^211^At‐AuNP‐SP > ^211^At‐AuNP > ^211^At.
	^211^At‐AuNP‐PEG^[^ ^55^ ^]^	In vitro, evaluated on C6 (glioma) and PANC‐1 (pancreatic) cells with cytotoxic effects for large ^211^At‐AuNP‐PEG (120 nm). In vivo, single intratumoral injection to C6 and PANC‐1 tumor‐bearing mice, suppression of tumor growth with 5 nm ^211^At‐AuNP‐PEG.
	^211^At‐AuNP‐PEG^[^ ^56^ ^]^ ^211^At‐AuNP‐H16 ^211^At‐AuNP‐RGD	In vitro, evaluated on PANC‐1, MDA‐MB‐231, and Hs 578Bst cells. Cellular internalization of ^211^At‐AuNP‐H16 (targeting tumor pH) and ^211^At‐AuNP‐RGD (targeting integrin α(v)β(3)) > ^211^At‐AuNP‐PEG. In vivo, ^211^At‐AuNP‐PEG intravenously injected to PANC‐1 xenografted mice, suppression of tumor growth.
	^211^At‐AuNStar‐PEG^[^ ^57^ ^]^	In vivo, intravenous injection (biodistribution) and intratumoral administration (therapeutic) to U‐87 MG xenografted mice, ≈85% tumor growth inhibition.
^225^Ac	^225^AcDOTA‐AuNP^[^ ^58^ ^]^	In vitro, decrease the viability of U‐87 MG cells in a concentration‐dependent manner. In vivo, intratumoral injection to U‐87 MG tumor‐bearing mice. Tumor growth index 2.4‐fold (8 d) and 3.9‐fold lower (22 d), compared to normal saline.
^198^Au	GA‐^198^AuNP^[^ ^59^ ^]^	In vivo, single‐dose intratumoral injection of Gum Arabic (GA)‐^198^AuNP to mice bearing PC‐3 human prostate tumors, tumor regression and effective control over 30 days.
	GA‐^198^AuNP^[^ ^60^ ^]^	In vivo, investigation of the short‐term safety profile of GA‐^198^AuNP intralesionally injected to dogs with spontaneously occurring prostatic cancers. No therapeutic assays.
	EGCg‐^198^AuNP^[^ ^61^ ^]^	In vivo, epigallocatechin‐gallate (EGCg) acted as a reducing agent to produce AuNP and ligand targeting Laminin67R receptors overexpressed in prostate tumor cells. EGCg‐^198^AuNP injected intratumorally to PC‐3 xenografted mice, 80% reduction of tumor volumes after 28 days.
	MGF‐^198^AuNP^[^ ^62^ ^]^	In vivo, Mangiferin (MGF) acted as a reducing agent to produce AuNP and targeting ligand thanks to tumor glucose metabolism. MGF‐^198^AuNP injected intratumorally to PC‐3 xenografted mice, 5‐fold reduction of tumor volumes after 3 weeks.
	^198^AuNP‐RGD^[^ ^63^ ^]^	In vitro, cell binding specificity toward B16F10 (murine melanoma) cell line. In vivo, intravenous injection, 8.7% ID g^−1^ uptake in the tumor 4 h postinjection, decrease in uptake (2.9% ID g^−1^) when RGD was co‐injected.
	^198^AuNP‐PAMAM‐Folic Acid^[^ ^64^ ^]^	In vitro, polyamidoamine dendrimer (PAMAM) modified with folic acid (for targeting overexpressed folate receptors), PEG, and ^198^AuNP. Evaluated on 4T1 cell line.
	^198^AuNP‐PEG‐RGD^[^ ^65^ ^]^	In vitro, ^198^AuNP‐PEG‐RGD targeting U‐87 MG cells showed accumulation and cytotoxic potency. Of note, ^197^AuNP activated by neutron irradiation to produce ^198^AuNP.
	^198^AuNP‐PEG^[^ ^66^ ^]^	In vivo, chemoradiotherapy, administered by convection‐enhanced delivery to U‐87 MG (glioblastoma)‐tumor‐bearing mice combined with oral administration of temozolomide. Median survival = 28 d (nontreated), 35 d (^198^AuNP‐PEG), 44 d (TMZ), and 54 d (^198^AuNP‐PEG/TMZ). Of note, ^198^AuNP produced by neutron reduction of HAuCl_4_.
	DOX^−198^AuNP‐Trastuzumab^[^ ^67^ ^]^	In vitro cytotoxic studies conducted on SKOV‐3 (ovarian cancer, HER2+) and MDA‐MB‐231 (breast cancer, HER2‐) cells, weaker cytotoxic effects on MDA‐MB‐231. In vivo, SKOV‐3 xenografted mice intratumorally injected, 82% reduction of tumor growth.
	Micelles‐^198^AuNP‐^223^Ra^[^ ^68^ ^]^	In vitro, Pluronic F127 micelles encapsulating both ^223^RaCl_2_ (α emitter) and ^198^AuNP evaluated on SaOS‐2 human osteosarcoma cell line.
	SPION‐^198^Au‐PEG^[^ ^69^ ^]^	In vitro, superparamagnetic iron oxide nanoparticles covered with a layer of ^198^Au and PEG tested on HepG2 cells (hepatocellular carcinoma). SPION magnetic hyperthermia properties investigated but not for synergistic cytotoxic effects.
^131^I	PEG‐AuNP‐Cetuximab‐^131^I^[^ ^70^ ^]^	In vitro, PEG‐AuNP‐Ctx‐^131^I showed a dose‐dependent cytotoxicity response on A549 human lung cancer cells. In vivo, A549‐tumor uptake PEG‐AuNP‐Cetuximab‐^131^I after intravenous injection by SPECT/CT (mice model) but no therapeutic assay conducted.
	^131^I‐PEI‐AuNP‐BmK CT^[^ ^71^ ^]^	Polyethylene imine modified with BmK CT (glioma‐targeting peptide), AuNP and ^131^I. In vitro, C6 cell viability decreased with increased radioactivity. BmK CT permitted higher uptake. In vivo inhibition of tumor growth by intravenous injection to C6 tumor‐bearing mice.
	^131^I‐PEI‐AuNP‐CTX^[^ ^72^ ^]^	In vitro/in vivo. Polyethylene imine modified with CTX (chlorotoxin, a glioma‐targeting peptide), AuNP, and ^131^I cross the blood–brain barrier after intravenous injection into rats and target glioma cells. Therapeutic efficacy not assessed.
	^131^I‐AuNRods‐PEG^[^ ^73^ ^]^	In vitro/in vivo. Intratumoral injection to mice bearing MCF‐7 tumors. Combined radiotherapy/photothermal therapy suppressed the tumor and extended survival time.
	^131^I‐AuNF‐PEG^[^ ^74^ ^]^	In vitro, ^131^I‐AuNFrameworks‐PEG combined with NIR laser irradiation resulted in lower 4T1 cells viability. In vivo, intravenous injection to 4T1 tumor‐bearing mice, led to accumulation in tumors. Combined radiotherapy and PTT > PTT or radiotherapy alone.
	^131^I‐PEI‐AuNP‐APAS^[^ ^75^ ^]^	In vitro/in vivo, polyethylene imine modified with AuNP, ^131^I and alkoxyphenyl acylsulfonamide (APAS) as pH‐responsive unit favoring cellular internalization. SPECT/CT imaging and radiotherapeutic effect in C6‐xenograft murine model (intravenous injection).
^125^I	^125^I‐AuNP‐RGD^[^ ^76^ ^]^	In vivo, intravenous injection to NCI‐H446 tumor‐bearing mice. Tumor growth inhibition by ^125^I‐AuNP‐RGD > nontargeted AuNP/external radiotherapy. ^125^I served as therapeutic factor and radiotracer.
	^125^I‐AuNP‐PEG^[^ ^77^ ^]^	In vitro, ultrasmall AuNP (1.9 nm) combined with ^125^I accumulated in cell nucleus, promoted U‐87 human glioblastoma cell killing (2D/3D tumor models).
	AuNP‐PDA‐^125^I^[^ ^78^ ^]^	In vitro/in vivo. Submicrometer AuNP covered with polydopamine as embolism beads. Intratumoral injection of AuNP‐PDA‐^125^I beads allowed radiosensitization of MHCC97H hepatocellular carcinoma cells.
^111^In	^111^In‐AuNP‐Trastuzumab^[^ ^79^ ^]^	In vitro, target HER2 receptors, ^111^In‐AuNP‐trastuzumab selectively interacted with SK‐BR‐3 (HER2++) and MDA‐MB‐361 (HER+) cells and was more potent than ^111^In‐AuNP. In vivo, intratumoral injection to HER2‐positive MDA‐MB‐361 xenografted murine model.
	^111^In‐AuNP‐EGF^[^ ^80^ ^]^	In vitro, target Epidermal Growth Factor‐positive cancer cells, evaluated on MDA‐MB‐468 (EGFR++) and MCF‐7 (EGFR+) cells. ^111^In‐AuNP‐EGF hybrids more radiotoxic to MDA‐MB‐468.
	^111^In‐EGF‐AuNP‐PEG^[^ ^81^ ^]^	In vitro, targets EGFR‐positive cancer cells (MDA‐MB‐468). In vivo, intravenous injection to MDA‐MB‐468 xenografted mice. 2.8% ID g^−1^ in the tumor after 72 h. Tumor uptake enhanced by coadministration of free EGF blocking hepatic EGFR.
	^111^In‐AuNStar‐PEG^[^ ^82^ ^]^	In vitro/in vivo, used as surrogate of nonlabeled AuNS‐PEG to monitor biodistribution and tumor accumulation in SKOV‐3 xenografted mice (intravenous injection). AuNS‐PEG used in photothermal therapy.
	^111^In‐TiONT‐AuNP‐DOX^[^ ^83^ ^]^	In vitro/in vivo, used as surrogate of nonlabeled titanate nanotubes grafted with AuNP and docetaxel anticancer drug. Therapeutic effect assessed by intravenous injection of TiONT‐AuNP‐DOX to PC‐3 xenografted mice together with external radiotherapy.
^103^Pd	^103^Pd‐AuNSeed^[^ ^84^ ^]^	In vivo, prostate cancer, intratumoral injection to PC3‐tumor bearing mice by single‐dose. Tumor burden reduction after 5 weeks, no side effects on healthy organs with 95% of nanoseeds retained in tumors.
	^103^Pd‐^198^AuNP‐PEG^[^ ^85^ ^]^ ^103^Pd‐AuNP‐PEG	In vivo, prostate cancer, formulated in alginate and intratumorally injected to PC3‐tumor bearing mice. ^103^Pd‐AuNP‐PEG > ^103^Pd‐^198^AuNP‐PEG (colabeled nanoparticles caused necrosis to healthy tissues).

## 7 Conclusions and Outlook

This article reviews strategies for assembling and delivering ^177^Lu‐gold nanoparticle nanohybrid radiotherapeutics to tumor sites. Some “conventional” lutetium‐177 formulations are already available for the treatment, for example, of prostate‐specific membrane antigen (PSMA)‐positive metastatic castration‐resistant prostate cancer (^177^Lu‐PSMA‐617) or somatostatin receptor‐positive gastroenteropancreatic neuroendocrine tumors (^177^Lu‐DOTATATE). While some other formulations of lutetium‐177 are under advanced phases of clinical trials, the ^177^Lu‐gold nanohybrid forms seem not yet to be mature enough for translation into radiation oncology in humans as they still face numerous challenges: 1) There is no definite consensus on the preferred administration mode of the nanohybrids. Among the various delivery routes, local nanobrachytherapy could provide a suitable answer as it avoids, for the most part, the rapid capture of the nanoparticles by the reticuloendothelial system classically encountered upon systemic administration. Yet, intratumoral injection is invasive and adapted only to accessible and well‐defined tumors. 2) The potential medium‐ to long‐term retention of metal nanoparticles after administration could be problematic. Although gold is an intrinsically nontoxic metal, it is difficult to predict the exact fate of nanoparticles if they are trapped for a prolonged period in the body. This also raises the question of public acceptance toward nanotherapeutics. 3) Due to their size, which exceeds the renal filtration threshold (*≈*8 nm), current nanohybrids cannot be eliminated via the urinary tract, but rather by the more complex and slow hepatobiliary route, thus exposing the liver and spleen, among others, to some radiations. It is therefore essential to develop biocompatible nanoscale platforms with a hydrodynamic diameter suitable for renal excretion. 4) Achieving homogeneous dose deposition in the tumor is not trivial considering the colloidal nature of the nanohybrids. Possible strategies could involve the use of two radionuclides with different energies of emitted β^−^ particles (e.g., ^177^Lu and ^90^Y or ^67^Cu) or emitting β^−^ particles and short‐range α particles (e.g., ^177^Lu and ^225^Ac), or a combination of external beam and internal radiotherapy. Alternatives could also take advantage of focused ultrasonic waves to enhance distribution and homogenize delivery of ^177^Lu‐AuNP in the affected zone.^[^ ^51^ ^]^ 5) Many of the current approaches reported in the literature focus on targetable nanohybrids. In our view, more emphasis should be placed on developing nanoscale platforms with optimized physicochemical properties (size, stability, biocompatibility, and elimination route) rather than trying to make carriers hyper‐specific for a defined target by conjugation with multiple ligands.

In this respect, it would certainly be useful to adapt existing nanoscale carrier systems that have already been validated in nanomedicine practice with regard to their pharmacokinetic and biodistribution properties. Building on existing platforms should considerably improve the effectiveness of nanoradiotherapies and accelerate their potential transfer to the clinics. Further development is therefore still required before ^177^Lu‐AuNP nanohybrids can find their place in the radiotherapeutic arsenal for the fight against cancer.^[^ ^52^ ^]^

## Conflict of Interest

The authors declare no conflict of interest.
